# Identification and expression analysis of the GLK gene family in tea plant (*Camellia sinensis*) and a functional study of *CsGLK54* under low-temperature stress

**DOI:** 10.1038/s41598-024-63323-1

**Published:** 2024-05-30

**Authors:** Hongtao Wang, Fangfang Xu

**Affiliations:** 1 Key Laboratory of Landscape Architecture, College of Landscape Architecture, Nanjing Forestry University, Nanjing, 210037 China; 2 Co-Innovation Center for Sustainable Forestry in Southern China, Nanjing Forestry University, Nanjing, 210037 China; 3 College of Forestry, Xinyang Agriculture and Forestry University, Xinyang, 464000 China

**Keywords:** *Camellia sinensis*, GLK family, Low-temperature stress, Genome-wide analysis, Functional validation, Computational biology and bioinformatics, Molecular biology

## Abstract

The Golden2-like (GLK) transcription factor family is a significant group of transcription factors in plantae. The currently available studies have shown that *GLK* transcription factors have been studied mainly in chloroplast growth and development, with fewer studies in abiotic stress regulation. In this study, all tea plant GLK transcription factors were identified for the first time in tea plants, and genome-wide identification, phylogenetic analysis, and thematic characterization were performed to identify 66 GLK transcription factors in tea plants. These genes are categorized into seven groups, and an amino acid sequence comparison analysis is performed. This study revealed that the structure of GLK genes in tea plants is highly conserved and that these genes are distributed across 14 chromosomes. Collinearity analysis revealed 17 pairs of genes with fragment duplications and one pair of genes with tandem duplications, and the analysis of Ka/Ks ratios indicated that most of the genes underwent negative purifying selection. Analysis of promoter *cis*-elements revealed that the promoters of tea plant GLK genes contain a large number of *cis*-acting elements related to phytohormones and stress tolerance. In addition, a large number of genes contain LTR elements, suggesting that tea plant GLK genes are involved in low-temperature stress. qRT‒PCR analysis revealed that the expression of *CsGLK17*, *CsGLK38*, *CsGLK54*, *CsGLK11* and *CsGLK60* significantly increased and that the expression of *CsGLK7* and *CsGLK13* decreased in response to low-temperature induction. Taken together, the results of the transcription profile analysis suggested that *CsGLK54* may play an important regulatory role under low-temperature stress. The subcellular localization of *CsGLK54* was in the nucleus. Furthermore, *CsGLK54* positively regulated the transcription levels of the NbPOD and NbSOD genes under low-temperature stress, which led to an increase in POD and SOD enzyme activities and a decrease in MDA content. These findings provide valuable insights into the regulatory mechanism of low-temperature stress in tea plants.

## Introduction

There are two types of low temperatures: cold temperatures (0–15 °C) and freezing temperatures (< 0 °C). Different response mechanisms are induced in plants under low-temperature stress, which is a significant abiotic stress affecting plant growth and development^1^. A major environmental constraint limits plant development and crop productivity. Global climate change has exacerbated various adverse effects on agriculture, with low-temperature stress being the primary cause of damage to the cell membrane of plant cells. The cell membrane is crucially affected by low-temperature stress, leading to changes in its structure and stability, resulting in the leakage of ions and the generation of reactive oxygen species^2^. Additionally, low-temperature stress affects the cell state of the cell epidermis, particularly impacting cell photosynthesis and gas exchange^3^, as well as chloroplast development and synthesis^4^. Therefore, developing low-temperature-resistant plant varieties is a key challenge in plant breeding to mitigate the hazards caused by low-temperature stress.

Low-temperature tea plant research has been extensively conducted in recent years. Recent studies have confirmed that compared with freezing, low temperature induces more changes in the expression of genes encoding cold-responsive transcription factors^5^. Therefore, research on transcription factors has focused primarily on cold-responsive temperature treatments. The main families of transcription factors associated with plant cold resistance that have been studied include the AP2/ERF, bHLH, WRKY, MYB, NAC, bZIP, heat shock factor, GARS, and zinc finger protein families. Additionally, the GOLDEN2-LIKE (GLK) transcription factor is a member of the GARP family of MYB transcription factors^6^. Since its discovery as an important transcription factor in plant growth and development in 1998, research on GLK transcription factors has been increasing. GLK transcription factors play crucial regulatory roles in plant growth and development. For example, GLKs in Arabidopsis and rice exhibit functional redundancy in cell differentiation^7^. *SlGLK2* regulates the content of sugars and carotenoids in tomatoes^8,9^, while *BpGLK1* disrupts chloroplast development and regulates chlorophyll content in birch^10^. Moreover, GLK transcription factors also play significant regulatory roles in plant abiotic stresses. Silencing the *SlGLK29* gene reduces cold resistance in plants, while overexpression of *GhGLK1* in Arabidopsis plays a role in regulating drought and cold stress responses^11^. Furthermore, overexpression of the peanut GLK gene in Arabidopsis improves drought resistance by influencing morphological development and photosynthesis^12^. The GLK gene is also involved in plant ABA regulation and ubiquitin signaling mediation^13,14^.

Tea plant (*Camellia sinensis* (L.) O. Ktze), an evergreen woody species found in subtropical regions, primarily relies on its leaves for economic purposes. Low temperatures not only impact the geographical distribution of tea plants but also cause damage to their leaves, affecting tea quality and yield. The GLK transcription factor, a member of the GARP family, is characterized by two conserved structural domains: a GCT-box at the C terminal that is specific to GLK genes and a DNA binding domain (DBD). The hexapeptide sequence AREAEAA at the DBD is extremely conserved among the GARP family. This DBD exists in green algae and land plants, while the GCT-box is found in land plants only. The hexapeptide sequence AREAEAA at the DBD is extremely conserved among the GARP family. This DBD exists in green algae and land plants, while the GCT-box is found in land plants only^15^. Although numerous GLK transcription factors have been discovered to be involved in the response to low-temperature stress, in-depth studies on how these factors regulate abiotic stress in plants are lacking. In our study, we conducted a comprehensive analysis of tea plant GLK genes, including genome-wide identification, phylogenetic analysis, gene structure analysis, chromosomal localization, covariance analysis, Ka/Ks analysis, and gene expression pattern analysis. Additionally, we validated the function of the candidate gene *CsGLK54* under low-temperature stress through transient transformation. Our findings not only provide a preliminary understanding of the function of tea plant GLK genes and their potential roles but also identify *CsGLK54* as a positively regulated gene under low-temperature stress. This research not only sheds light on the regulatory mechanism of low-temperature stress but also offers valuable insights into potential functional genes involved in other plantae and plant improvement, thereby contributing significantly to breeding advancements.

## Materials and methods

### Material treatment

The experimental materials were sourced from the teaching practice garden of Nanjing Forestry University. For each treatment, 10 two-year-old Fuding Dabai tea plants with healthy growth and similar growth conditions were carefully selected. The growth conditions in the controlled environment of the artificial climate chamber were set as follows: a temperature of 24 ± 2 °C and humidity maintained at 65%. The plants that did not undergo any treatment at 4 °C were designated the control group (CK), while those subjected to 4 °C treatment for 7 days constituted the treatment group. Following treatment, there was a 7-day recovery period. After each treatment, the first three leaves were collected (mixed samples), consisting of one bud and three leaves for each treatment (mixed samples). Three biological replicates were maintained for each treatment. The collected samples were promptly frozen in liquid nitrogen and stored at – 80 °C for future use.

### Identification of GLK proteins in *C. sinensis*

The tea plant genomic data were obtained from the TPIA2 database (http://tpia.teaplants.cn/)^16^. A total of 40 amino acid sequences of Arabidopsis Golden2-like (GLK) transcription factors were downloaded from the Arabidopsis Information Resource (TAIR: http://www.arabidopsis.org/). Homology comparisons were conducted between the tea plant genome and Arabidopsis amino acid sequences using an E-value < 1.0e−5. In addition, we searched the tea plant genome by downloading the hidden Markov model Myb-DNA-binding (PF00249) with an E value < 1.0e−5 to the Pfam protein family data. The resulting gene protein sequences were then uploaded to PfamScan (https://www.ebi.ac.uk/Tools/pfa/pfamscan/) and SMART (http://smart.embl-heidelberg.de/) for retrieval with an E value < 1.0e−5 for searching, and the search results were used to determine whether the genes contained Myb-DNA-binding conserved structural domains. The physicochemical properties of tea plant GLK proteins, including amino acid number, molecular weight (MW), and isoelectric point (PI), were predicted using the online ProtParam tool (https://web.expasy.org/protparam/).

### Sequence comparison and phylogenetic analysis

Protein sequence comparison and construction of an evolutionary tree for the evolutionary analysis of tea plant *CsGLKs* were carried out. First, the amino acid sequences of the *CsGLKs* were sequenced using DNAMAN 6.0, and the conserved structural domains (50%) were labeled. Second, we constructed an evolutionary tree of the GLK genes of tea plants and *Arabidopsis thaliana* using MEGA X software via the maximum likelihood (ML) method and performed bootstrap tests (n = 1000)^17^, and the evolutionary trees were constructed via the Evolview (https://evolgenius.info//evolview-v2) online tool^18^.

### Analysis of *C. sinensis* GLK motifs and promoter *cis*-acting elements

Motif prediction of tea plant GLK amino acid sequences using the online tool MEME suite 5.5.5 (https://memesuite.org/meme/) (MEME parameter settings: number of Motifs is 10, the rest of the parameters are default)^19^. The 2.0 kb promoter sequence upstream of the *CsGLKs* was extracted using TBtools V2.001, and the online tool PlantCARE (http://bioinformatics.psb.ugent.be/webtools/plantcare/html/) was utilized to identify the gene homeopathic action elements. The results were visualized using TBtools V2.001 software.

### Chromosome localization and collinearity analysis

The identified genes were filtered and organized by the provided information, the identified *CsGLK* genes were visualized and localized on the tea plant chromosomes using TBtools, and synonymous analysis was performed using TBtools V2.001^20^.

### Gene expression profiling and quantitative real-time PCR analysis

The FPKM values generated from the RNA-Seq data (Table S1) were obtained from the public database of Anhui Agricultural University (http://tpia.teaplant.org/index.html), and TBtools was used to generate heatmaps. RNA extraction was performed using a polysaccharide polyphenol plant RNA extraction kit (Beijing Huayueyang Biotechnology Co., Ltd.), and the primers (Table S2) used were derived from previous studies^21,22^. The real-time fluorescence quantification system included 1 μL of cDNA template, 10 μL of SYBR Premix ExTaq (Takara, Kyoto, Japan), 2 μL of specific primers, and 7 μL of ddH_2_O. The PCR heat cycle parameters were as follows: 95 °C for 5 min, 95 °C for 20 s, 60 °C for 20 s, and 72 °C for 10 s for 45 cycles. Real-time fluorescence quantitative PCR analysis of cDNA from different node samples was performed using a Bio-Rad CFX96 fluorescence quantification system (Bio-Rad, Hercules, CA, USA). Nbactin and CsPTB-RT were used as specific primers for internal control of tobacco and tea plants to normalize gene expression. The relative expression of genes was determined and analyzed by the 2^−ΔΔCT^ method with three biological replicates for each sample.

### Subcellular localization

Specific primers were used to amplify *CsGLK54* without the stop codon (Table S2), and the binary vector GFP (pCAMBIA1300-35S: CDS-GFP) was inserted. A 35-day-old tobacco leaf was injected for transient expression mediated by Agrobacterium using the 35S-GFP vector as a control^23^. 2 days later, the transformed leaves were stained with 5 μM DAPI for 15 min in the dark, and the fluorescence signal was observed using an LSM 710 laser confocal microscope (Zeiss, Germany).

### Verification of *Nicotiana* benthamiana transient conversion

The instantaneous conversion function validation method was described previously^24,25^. Full-length *CsGLK54* was ligated into the pSAK277 vector. The sequences of primers used for expression vector construction can be found in Table S2. The recombinant vector was transformed into *Agrobacterium rhizogenes* strain GV3101 using chemical methods. Transient expression assays were performed in tobacco plants cultured under specific conditions: 14 h of light, 10 h of darkness, 25 °C temperature, and 70% relative humidity. Plants with good growth status were selected for injection. Agrobacterium containing pSAK277-CsGLK54 was cultured overnight until it reached an OD600 of approximately 1.2. The organisms were then collected at 5000 rpm/min for 5–10 min. The resuspension solution (10 ml) used for the infiltration solution contained MES (working concentration: 10 mM), MgCl_2_ (working concentration: 10 mM), and acetosyringone (working concentration: 100 µM). The OD600 of the resuspended bacterium was adjusted to approximately 0.8 before use. The infiltration solution was left to stand for 3–4 h after preparation. After injection, the cultures were cultured under normal conditions for 2–3 days, followed by treatment at 4 °C for 12 h. For each treatment, three biological replicates of tobacco were injected, and the samples were mixed and sampled.

### Analysis of physiological indicators

The relevant physiological indices, such as conductivity, were determined via the use of a perforator to punch holes for each replicate index. The parameters were mixed with 10 mL of distilled water, added to the test tubes, and soaked at room temperature for 1 day (during which time the test tubes were shaken) to determine the conductivity value C1 using a conductivity meter. The samples were then placed in a water bath at 100 °C for 15 min, after which they were removed and cooled to room temperature to determine the conductivity value C2. Distilled water was used as a blank control.

A malondialdehyde (MDA) test kit was used for the determination of malondialdehyde. MDA is condensed with thiobarbituric acid (TBA) to produce a red product, which has a maximum absorption peak at 532 nm, and the content of lipid peroxide in the sample can be estimated after colorimetry. At the same time, the absorbance at 600 nm was measured, and the difference between the absorbances at 532 nm and 600 nm was used to calculate the content of MDA. At the same time, the absorbance at 600 nm was measured, and the difference between the absorbance at 532 nm and 600 nm was used to calculate the content of MDA.

The enzyme activity was determined with reference to a previous method^26–28^, and the method was improved by adding 0.2 g of liquid nitrogen-ground leaf powder to a test tube, adding 5 ml of 0.05 mol/L (pH 7.8) phosphate buffer and mixing well, and centrifuging for 20 min (8500 rpm/min) to extract the crude enzyme extracts. The activity of superoxide dismutase (SOD) was determined by using the aziridinium blue tetrazolium (NBT) method. The dark treatment and light acclimatization treatments were carried out separately. The dark treatment was used as a blank, and the absorbance at 560 nm was measured in the light control tube and other tubes. The peroxidase (POD) activity was determined by the guaiacol method. One milliliter of 0.05 mol/L guaiacol was added to the crude enzyme extract, and the absorbance at 470 nm was measured by rapidly shaking the reagent and mixing it well. The absorbance was recorded every 30 s for a total of three times. Catalase (CAT) activity was determined by the ultraviolet absorbance method. Then, 0.3 mL of 0.1 mol/L hydrogen peroxide solution was added to the crude enzyme extract, and 0.2 mL of enzyme solution (the control was 0.2 mL of phosphate buffer, pH 7.8) was added. The absorbance at 240 nm was determined by shaking the reagent quickly and mixing the reagent, and the absorbance was recorded once every 30 s for a total of 3 min.

### Data analysis

The data were analyzed for data processing and significant differences using SPSS 22.0 and plotted using GraphPad Prism 8.0. The data obtained were shown below mean ± standard errors (SE). Data were all analyzed using Student’s *t*-test and one-way ANOVA. In the representation of the results, different letters as well as asterisks were used to indicate that the differences were statistically significant (*P* < 0.05).

### Ethics approval and consent to participate

These plant materials do not include any species at risk of extinction. We declare that all the experimental plants were collected with permission from local authorities of the agricultural department and the plant materials used in the experiment. We complied with relevant institutional, national, and international guidelines and legislation for plant studies.

## Results

### Identification of GLK genes in *C. sinensis*

In this study, a total of 66 tea plant GLK genes were identified in the genome of the tea plant CSS (cv. Shuchazao), and evolutionary tree analysis revealed that the family members could be divided into seven subfamilies. We also predicted and analyzed the basic physical and chemical properties of the genes. The results showed that the length of the amino acid sequences ranged from 137 aa (*CsGLK50*) to 686 aa (*CsGLK13*), the predicted theoretical isoelectric points ranged from 5.26 (*CsGLK49* and *CsGLK66*) to 10.05 (*CsGLK3*), the length of the open reading frames ranged from 411 bp (*CsGLK50*) to 2,058 bp (*CsGLK13*), and the predicted molecular weight was 15.52 kDa (*CsGLK50*) ~ 74.68 kDa (*CsGLK13)* (Table S3).

### Evolutionary analyses

**Figure 1:**
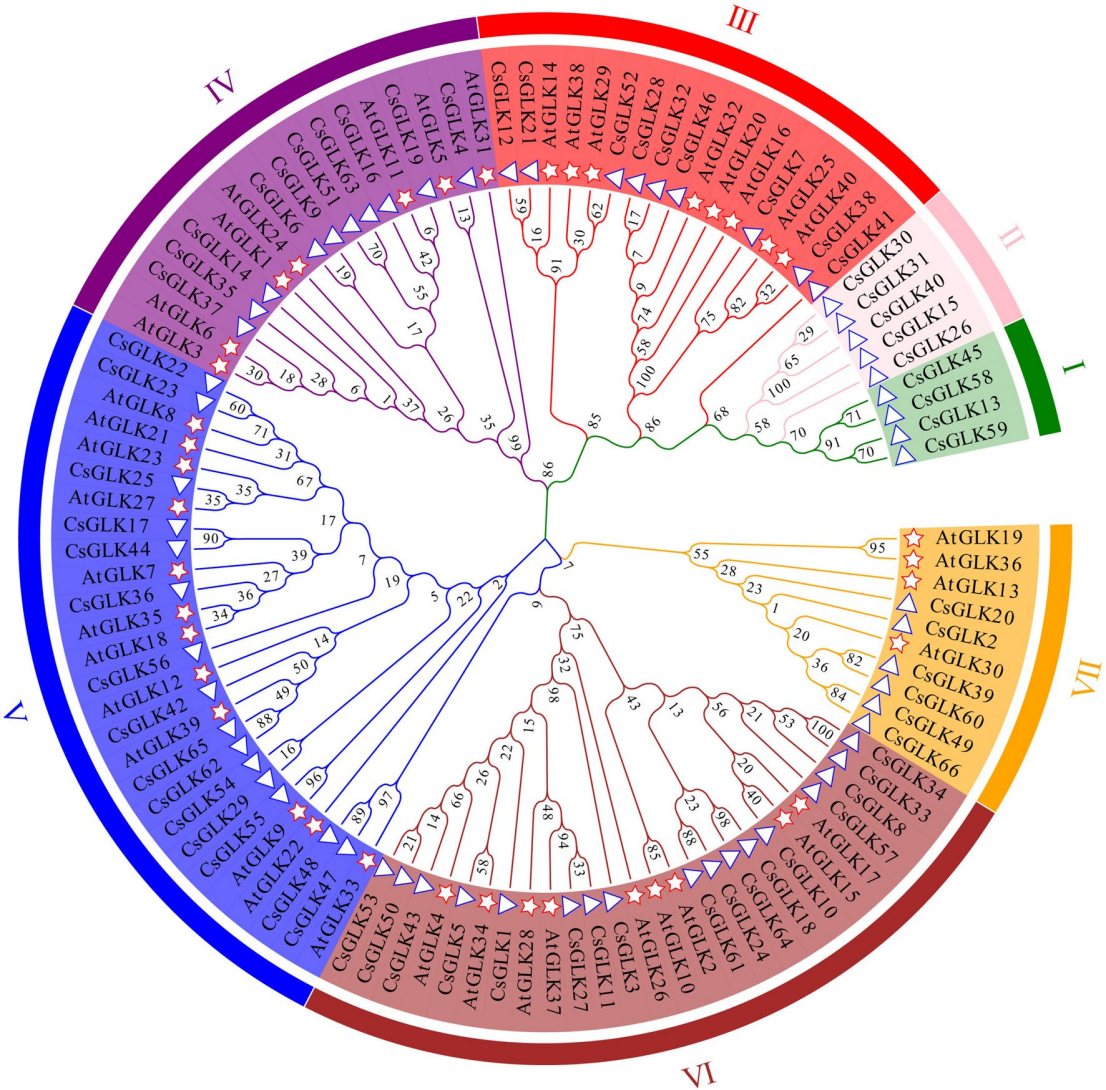
Phylogenetic analysis of GLK genes in *C. sinensis* and *Arabidopsis thaliana;* the pentagrams represent *Arabidopsis thaliana*, and the triangles represent *C. sinensis.*

To study the evolutionary relationships among the *CsGLK* genes, we constructed a maximum likelihood evolutionary tree using *Arabidopsis thaliana* (40) and *C. sinensis* (66) GLK protein sequences as a reference. The results showed that the evolutionary tree was divided into seven subfamilies (I, II, III, IV, V, and VI/VII), and the different subfamilies are represented by seven different colors in the figure (Fig. 1). The evolutionary analysis revealed that the GLK proteins in tea plants were distributed in various groups, while the individual genes showed high homology with *the* GLK gene protein sequences in *Arabidopsis thaliana*. The results not only indicated that the homologous genes might have certain functional similarities but also provided information about the direct homology of GLK proteins in different species.

### Conserved motif and gene structure analysis of the CsGLK proteins

**Figure 2:**
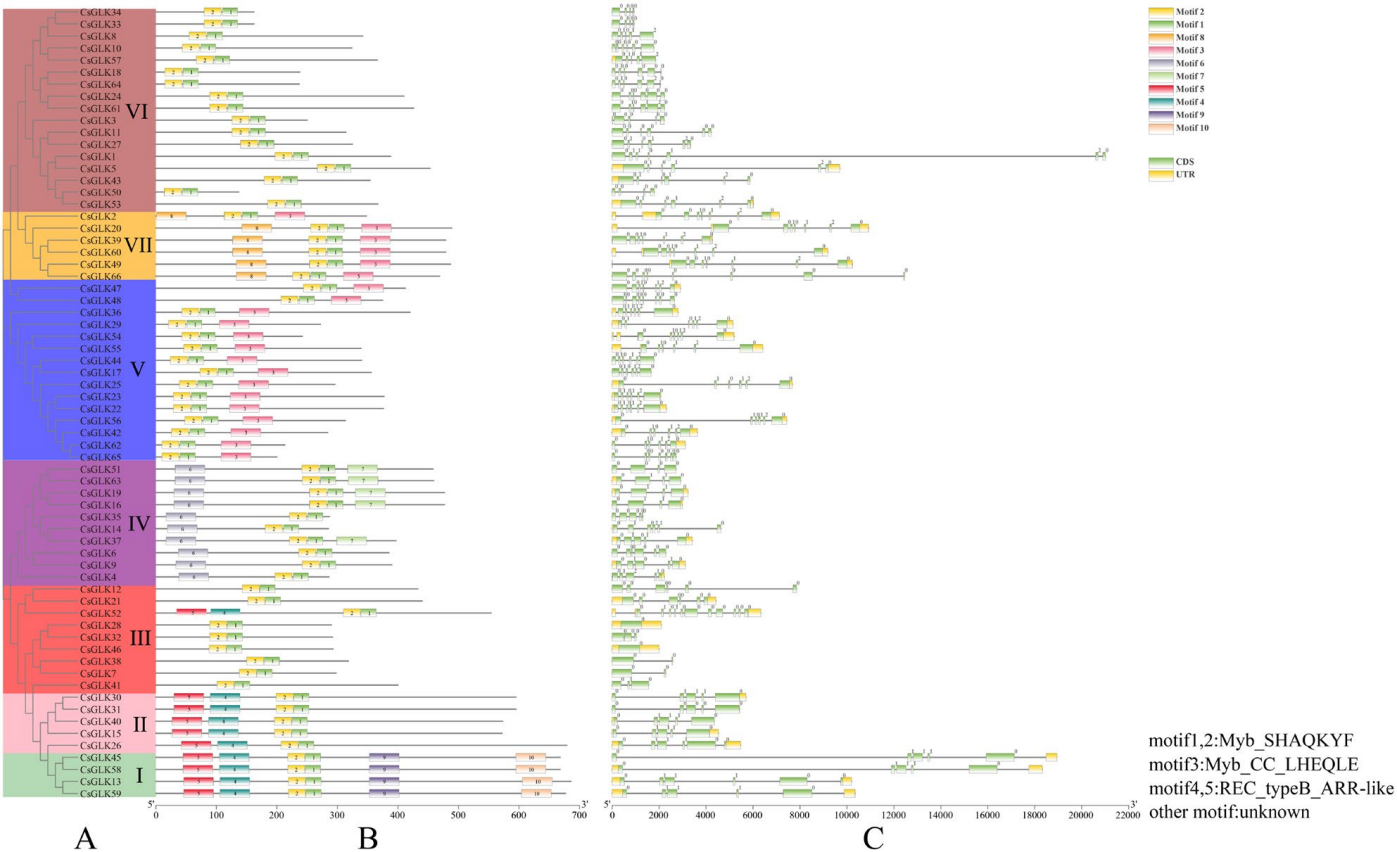
Phylogenetic evolutionary tree (**A**), conserved motif (**B**) and gene structure (**C**) analysis of the *C. sinensis* GLK gene family.

To analyze the evolutionary relationships among the tea plant GLK genes, we used 66 CsGLK protein sequences to construct a phylogenetic evolutionary tree (Fig. 2A). Based on motif distribution and evolutionary clustering relationships, the members were classified into seven groups. The conserved motifs were analyzed using the online MEME tool (Fig. 2B), and their amino acid sequences were uploaded to NCBI-CDD for functional annotation. Motifs 1 and 2 were defined as Myb-SHAQKYF, motif 3 as Myb_CC_LHEQLE, and motifs 4 and 5 as REC_typeB_ARR-LIKE (Fig. 2). The other motifs were not functionally annotated. Additionally, the tea plant genes were categorized into 4 major groups based on motif classification (Table S4). Motif 1 and motif 2 constitute the conserved structural domains of tea plant GLK, and different subfamily members contain different motifs, indicating high differentiation during evolutionary development. Furthermore, we performed a structural analysis of the exons and introns of the tea plant GLK gene (Fig. 2C), revealing that all tea plant GLK genes contain introns ranging from 0 to 11 in number, suggesting a relatively complex gene structure.

**Figure 3:**
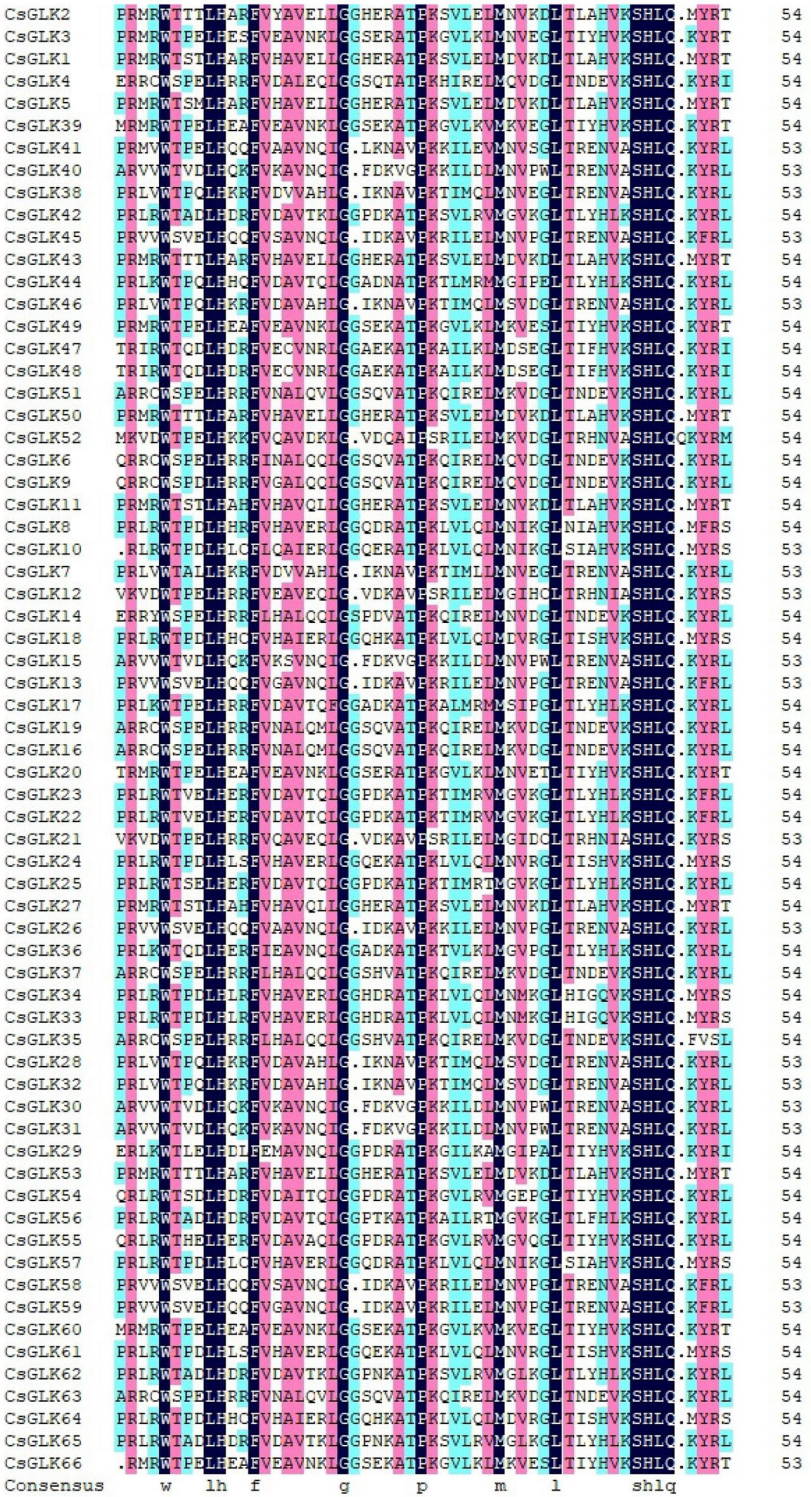
In the conserved regions of the *C. sinensis* GLK gene family, cyan represents ≥ 50%, pink represents ≥ 75%, and dark blue represents 100%.

To study the conserved structural domains of the tea plant GLK genes in greater depth, we used DNAMAN 6.0 to perform multiple alignment of the protein sequences of 66 *CsGLKs.* The results of the multiple alignment showed that in the tea plant GLK genes, all the genes contained two regions of putative DNA-binding structural domains with HLH structures. In addition, the first helix had the initial sequence of PELHRR, and the second helix contained highly conserved VK/VASHLQ sequences (Fig. 3), and this result was also verified in tobacco and citrus species. However, under the premise that the overall amino acid structure is highly conserved, there are partial deletions and alterations in the conserved amino acid sequences of some *CsGLK* genes, which suggests that the functions of these genes may be highly similar to those of the tea plant GLK genes, and at the same time, the functions of some of the genes may have diverged over the course of the evolutionary process.

### Chromosome localization, gene duplication and evolutionary analysis

**Figure 4:**
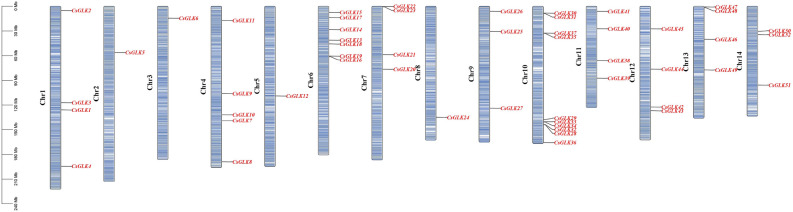
Chromosome localization analysis of the *C. sinensis* GLK gene family.

**Figure 5:**
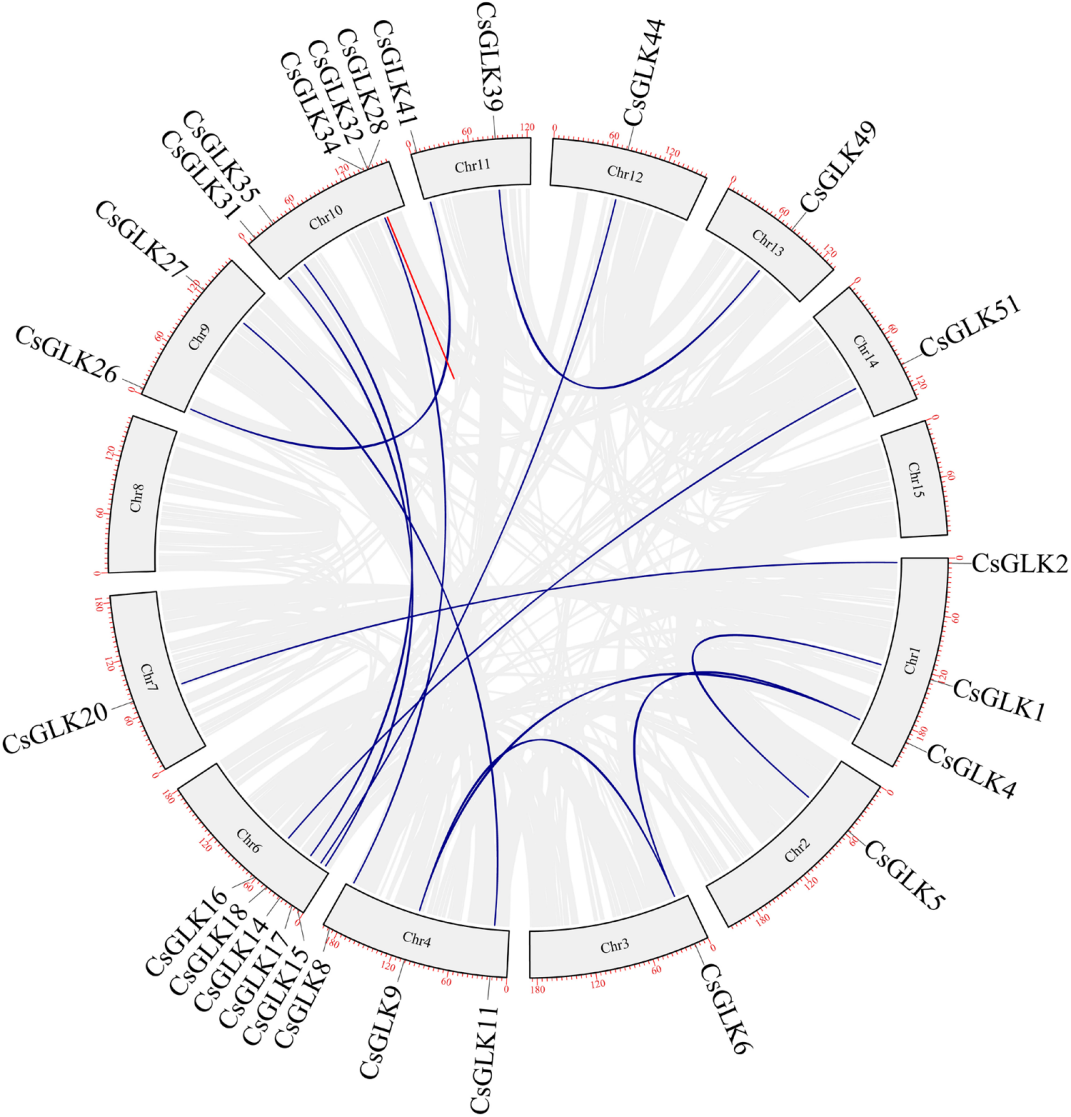
Collinearity analysis of the *C. sinensis* GLK gene family; red indicates tandem replication events, and blue indicates fragment replication events.

52 genes were unevenly distributed on 14 chromosomes (Fig. 4), 14 genes were not localized on existing chromosomes due to incomplete genome assembly, 1–10 genes were distributed on each chromosome, the largest cluster of genes with five genes was formed on chromosome Chr10, followed by five and seven genes on chromosomes Chr04 and Chr06, respectively, and one GLK gene on each of Chr02, 03, 05 and 08 each had one GLK gene. We then analyzed the replication events of the tea plant GLK genes (Fig. 5), and among the *CsGLK* genes, a total of 17 pairs of genes were fragmentally replicated, and one pair of genes was tandemly replicated. The duplication events were mainly concentrated on Chr04, Chr06 and Chr10. To further analyze the evolutionary relationships between the genes, the Ka, Ks, and Ka/Ks ratios of the duplication events were calculated using DNASP 6.0 software, and the time of duplication occurrence was analyzed^29^. After eliminating the data with more synonymous mutation sites and greater evolutionary distance, we obtained information on 16 pairs of duplication events; interestingly, all gene pairs had Ka/Ks ratios less than 1, and the evolutionary time ranged from 0.46 million to 100.25 million years (Table 1).

**Table 1:** Parameters and dates of the duplication events in the CsGLKs.

Gene name	Gene name	Ka	Ks	Ka/Ks	DATE (million years)
CsGLK1	CsGLK5	0.13	0.45	0.29	30.13
CsGLK4	CsGLK6	0.40	1.50	0.26	100.25
CsGLK4	CsGLK9	0.45	1.47	0.30	98.21
CsGLK2	CsGLK20	0.17	0.59	0.29	39.36
CsGLK32	CsGLK28	0.03	0.04	0.60	2.87
CsGLK34	CsGLK8	0.22	0.61	0.36	40.35
CsGLK31	CsGLK15	0.12	0.39	0.32	26.04
CsGLK35	CsGLK14	0.23	0.68	0.34	45.52
CsGLK39	CsGLK49	0.17	0.34	0.49	22.62
CsGLK41	CsGLK26	0.51	1.92	0.27	127.72
CsGLK44	CsGLK17	0.19	0.63	0.30	41.67
CsGLK49	CsGLK66	0.03	0.06	0.45	3.72
CsGLK51	CsGLK16	0.13	0.54	0.24	35.73
CsGLK6	CsGLK9	0.20	0.55	0.37	36.34
CsGLK11	CsGLK27	0.14	0.37	0.38	24.57
CsGLK18	CsGLK64	0.00	0.01	0.00	0.46

Note: T = Ks/2λ is the formula for calculating the date of the repeated event, λ is the estimated clock sample rate of synonymous substitution in dicotyledons, λ = 1.5 × 10^–8^ substitutions/synonymous sites/year.

### Analysis of promoter *cis*-acting elements

**Figure 6:**
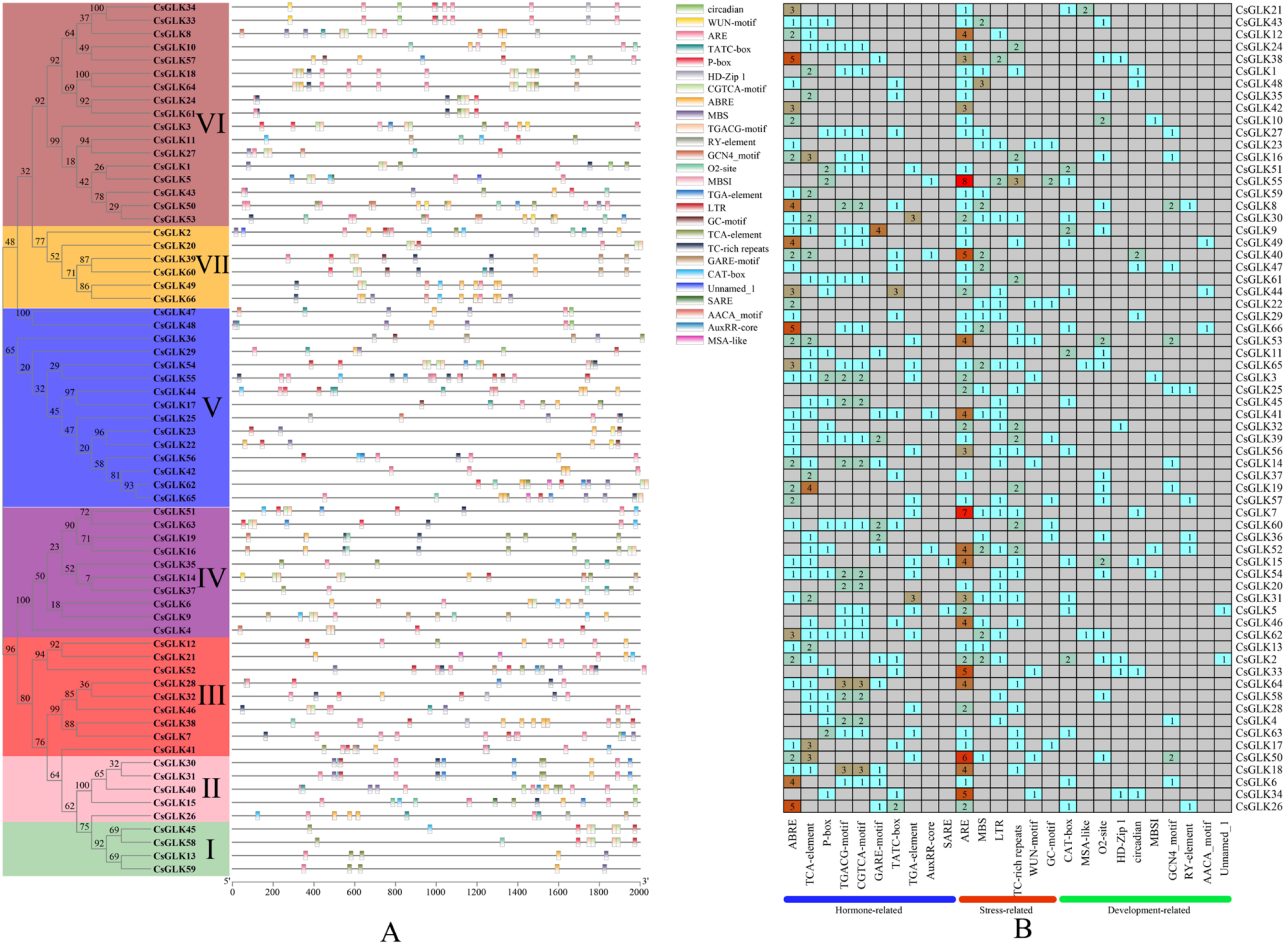
Cis-element analysis of *C. sinensis* GLK gene promoters. The binding sites in the promoter region are represented by boxes of different colors (**A**), and the graph shows the number of binding sites (**B**).

To further analyze the potential response mechanisms of tea plant GLK genes in response to cold stress, 66 *cis*-acting elements within the promoter 2 kb upstream of tea plant GLK genes were analyzed. The results revealed three types of *cis*-acting elements, namely, stress-related (anaerobic induction, hypoxia-specific induction, drought induction, low temperature, and wound response), development-related (endosperm expression, seed-specific regulation, meristematic tissue expression, regulation of zein metabolism, control of circadian rhythms, and downregulation of the expression of photosensitizing pigments), and hormone-related (gibberellin response, abscisic acid response, MeJA response, and salicylic acid response) homeostatic components (Fig. 6A).

The results revealed that 53 genes contained one or more stress-responsive *cis*-acting elements (AREs), 24 genes contained low-temperature-responsive elements (LTR), and 43 genes contained abscisic acid-responsive *cis*-acting elements (ABREs) (Fig. 6B). Several *cis*-acting elements, ABREs, MBSs, G-boxes, W-boxes, and LTRs, play important roles in the regulation of downstream gene expression after plant stress, especially LTRs, which are low-temperature-responsive *cis*-acting elements. These findings suggest a potential response mechanism for the GLK gene in tea plants under low-temperature stress. Combined with the presence of other *cis*-acting elements involved in development and hormones, these findings suggest that in tea plants, GLK genes are involved in physiological developmental processes and hormone induction.

### Expression analysis of the CsGLK gene under low-temperature stress

**Figure 7:**
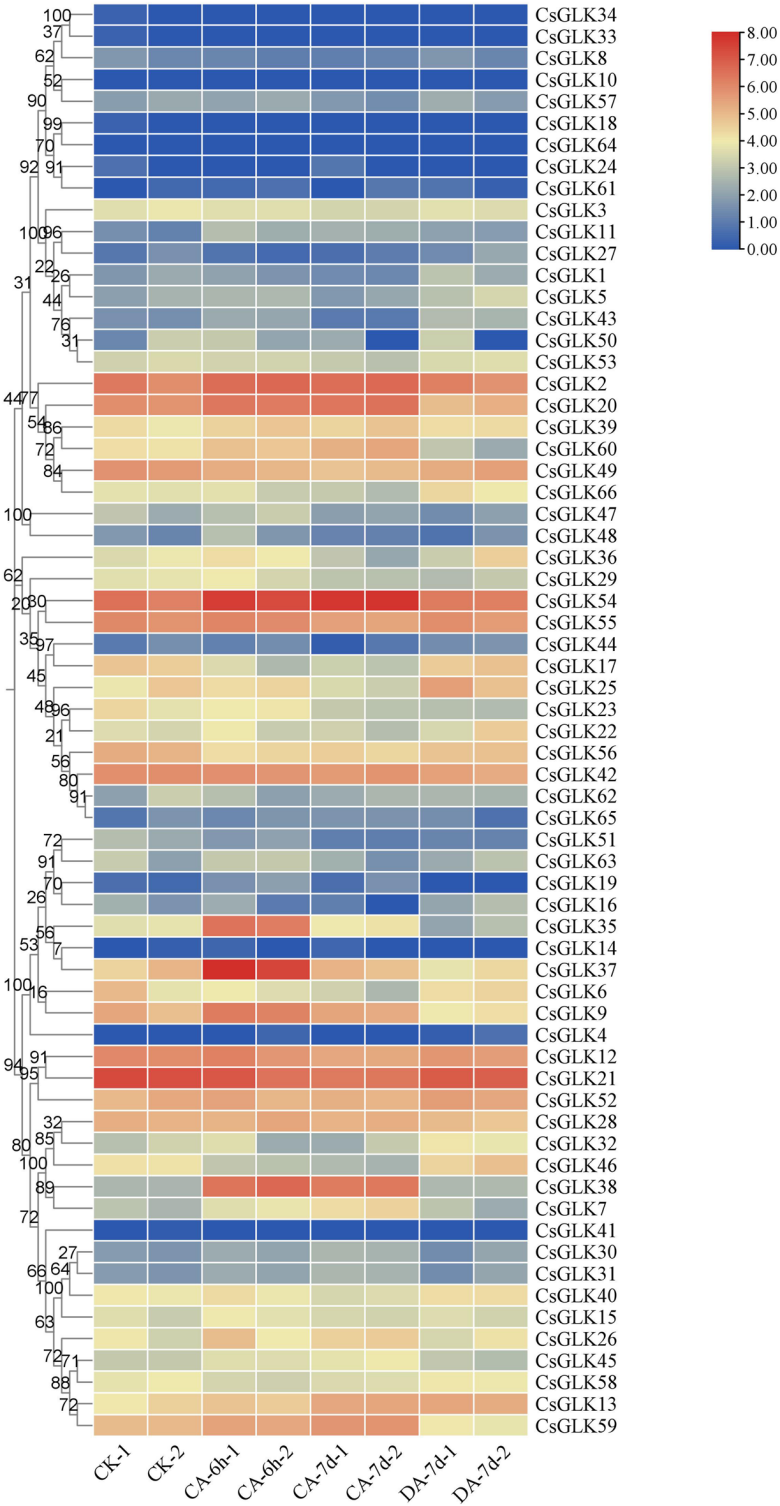
FPKM expression profile of the *C. sinensis* GLK gene after low-temperature stress and room temperature recovery based on RNA-Seq data. The data were subjected to log2 processing. The color scale on the left side represents the relative expression values, CK: control, CA: 10 °C treatment, DA: room temperature recovery.

The FPKM values obtained for 66 tea plant *CsGLK* genes under low-temperature stress were used to construct a heatmap, and the expression patterns of the tea plant *CsGLK* genes under low-temperature stress were subsequently explored. The results showed that the expression of most genes differed under low-temperature stress, among which 12 genes were not expressed during the treatment stages, and these genes were hardly expressed under low-temperature stress, probably because they were not involved in low-temperature stress, whereas genes whose expression differed might play important regulatory roles in the low-temperature response under low-temperature stress. In addition, at different stages of treatment, we noticed that most of the expressed *CsGLK* genes were differentially expressed after 6 h of treatment, and the difference in expression between before and after treatment was high (Fig. 7). The expression of some of the genes also diverged at 7 d of treatment, with some genes showing a gradual increase in expression and some showing a decrease in expression under prolonged low-temperature stress, whereas the difference in gene expression was similarly large after the recovery culture, with most of the genes expressed at the same level as the normal one after the recovery culture. The expression of genes mostly differed from the normal level. In addition, we found that the genes in part VI were hardly expressed or were expressed at a lower level, and similar expression patterns were observed for the genes expressed in parts I and II. The complexity of the results reinforces our conviction that *CsGLKs* may play an important regulatory role under low-temperature stress, and second, the expression patterns of structurally similar genes were also more similar, suggesting that gene similarity is also similar in terms of functional expression. To preliminarily determine which genes may play a role in low-temperature stress, combined with expression pattern analysis, we used FPKM > 10 as the reference standard and excluded genes that did not meet the standard. At the same time, we performed log2-fold change treatment to compare the expression changes of genes before and after treatment to select genes with large differences before and after changes and at the same time to require the genes to return to the normal level of expression after restoration of the treatment. After eliminating the data that did not meet the criteria, we initially identified *CsGLK7*, *CsGLK17*, *CsGLK38*, and *CsGLK54* as the candidate genes and found that the expression of *CsGLK17* and *CsGLK36* decreased at 7 d of treatment and that the expression of *CsGLK7*, *CsGLK38*, and *CsGLK54* increased after treatment, which showed that these genes might mediate the low-temperature response process of tea plants under low-temperature stress.

**Figure 8:**
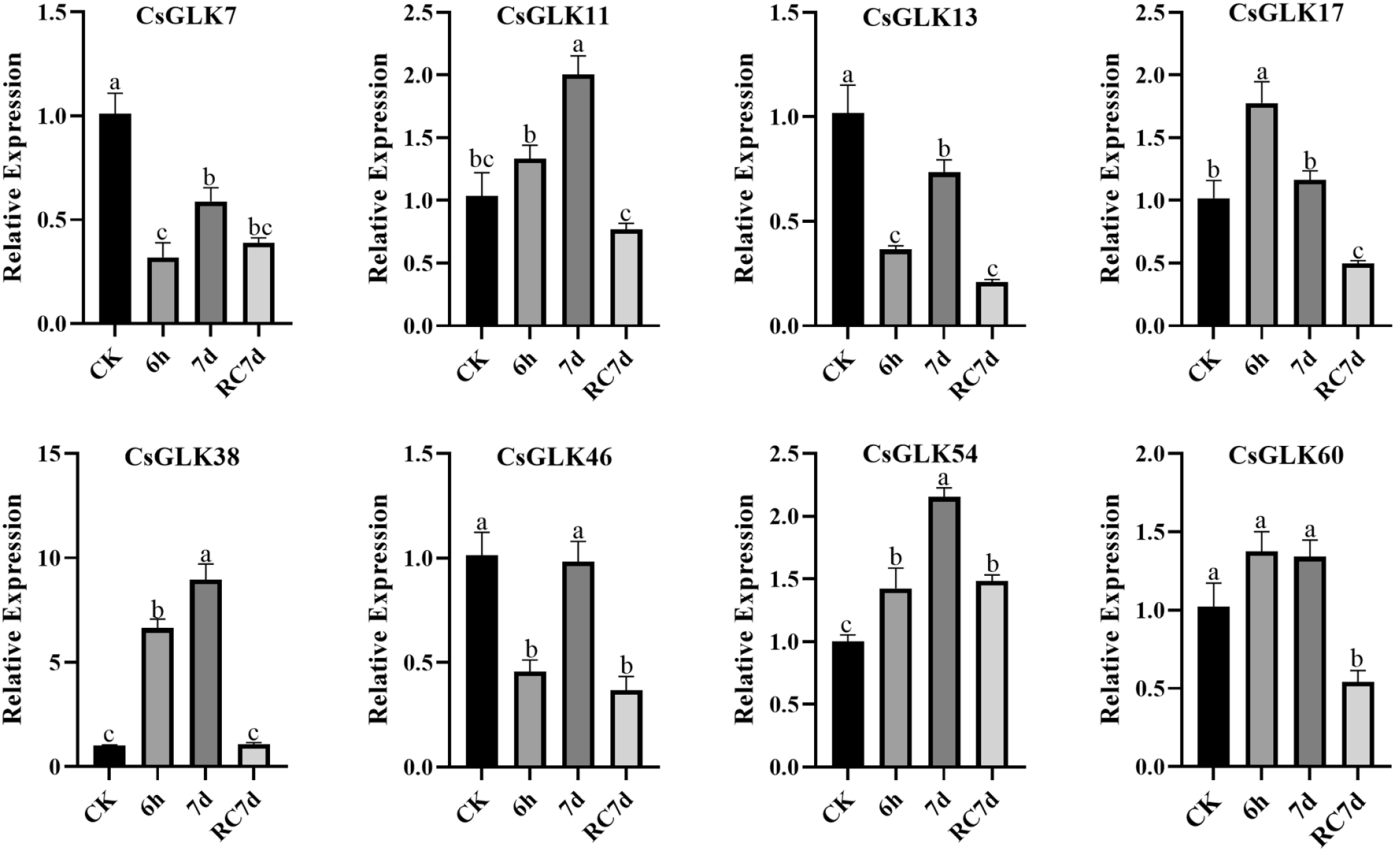
Relative expression levels of 8 selected GLK genes under cold stress. The bars represent the mean values of three replicates ± standard errors, and different letters indicate significant differences at different time points according to one-way ANOVA and least significant difference (LSD) tests (*P* < 0.05).

To further verify whether the expression of these genes was induced by low temperature, we selected four genes on the basis of the candidate genes for real-time fluorescence quantitative verification and found that the expression of *CsGLK17*, *CsGLK38*, *CsGLK54*, *CsGLK11* and *CsGLK60* was significantly increased by low-temperature treatment (Fig. 8), while the other genes showed significant decreases of varying degrees, of which *CsGLK38* and *CsGLK54* were induced to have the highest expression level (Table S5).

Among the candidate genes, the expression of the *CsGLK54* gene was greatest after both short-term and long-term low-temperature stress, and the expression also returned to the normal level after recovery. Meanwhile, real-time fluorescence quantification of *CsGLK54* after low-temperature treatment also indicated that the expression of *CsGLK54* was significantly increased by low-temperature induction.

### Subcellular localization of the CsGLK54 and functional validation of the CsGLK54

**Figure 9:**
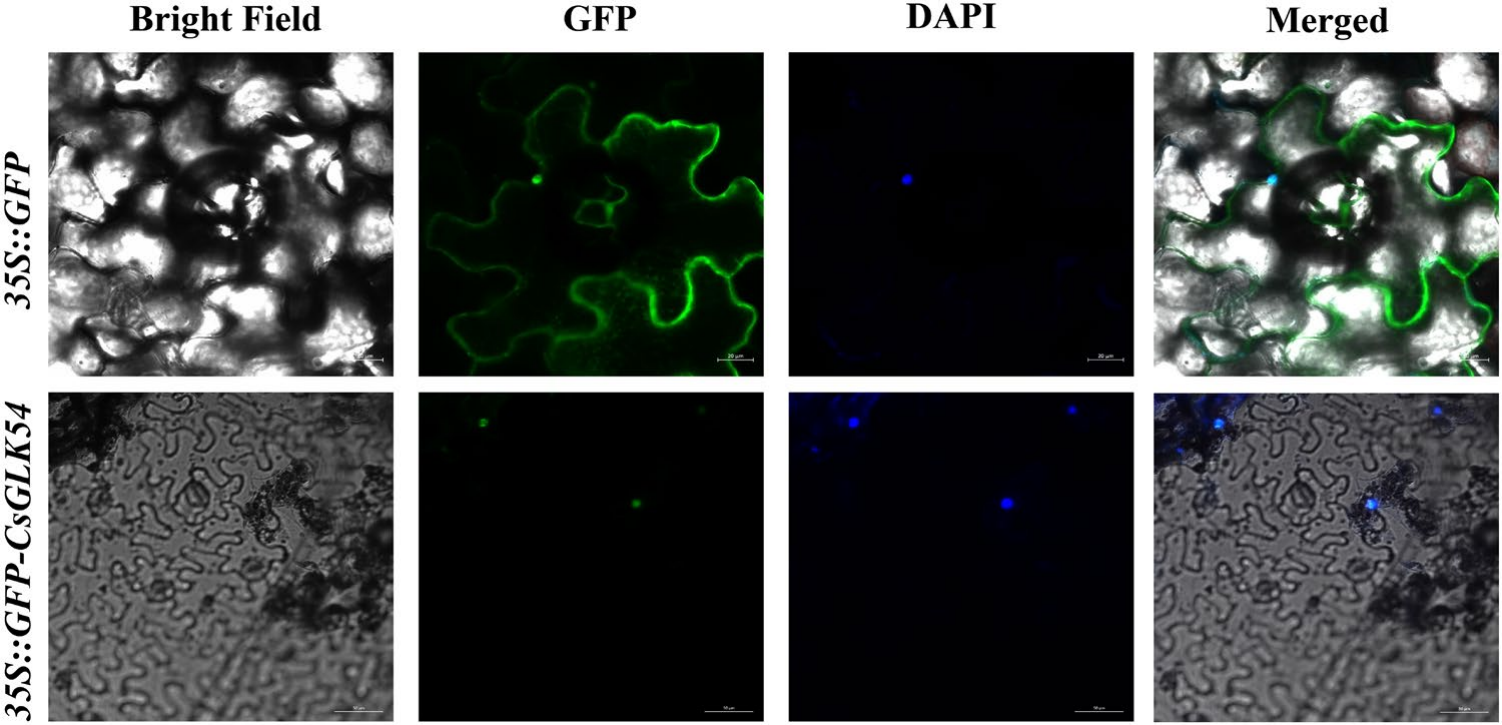
Subcellular localization of CsGLK54. Fluorescence signals from the nuclei of tobacco epidermal cells labeled with 4′,6-diamidino-2-phenylindole (DAPI).

**Figure 10:**
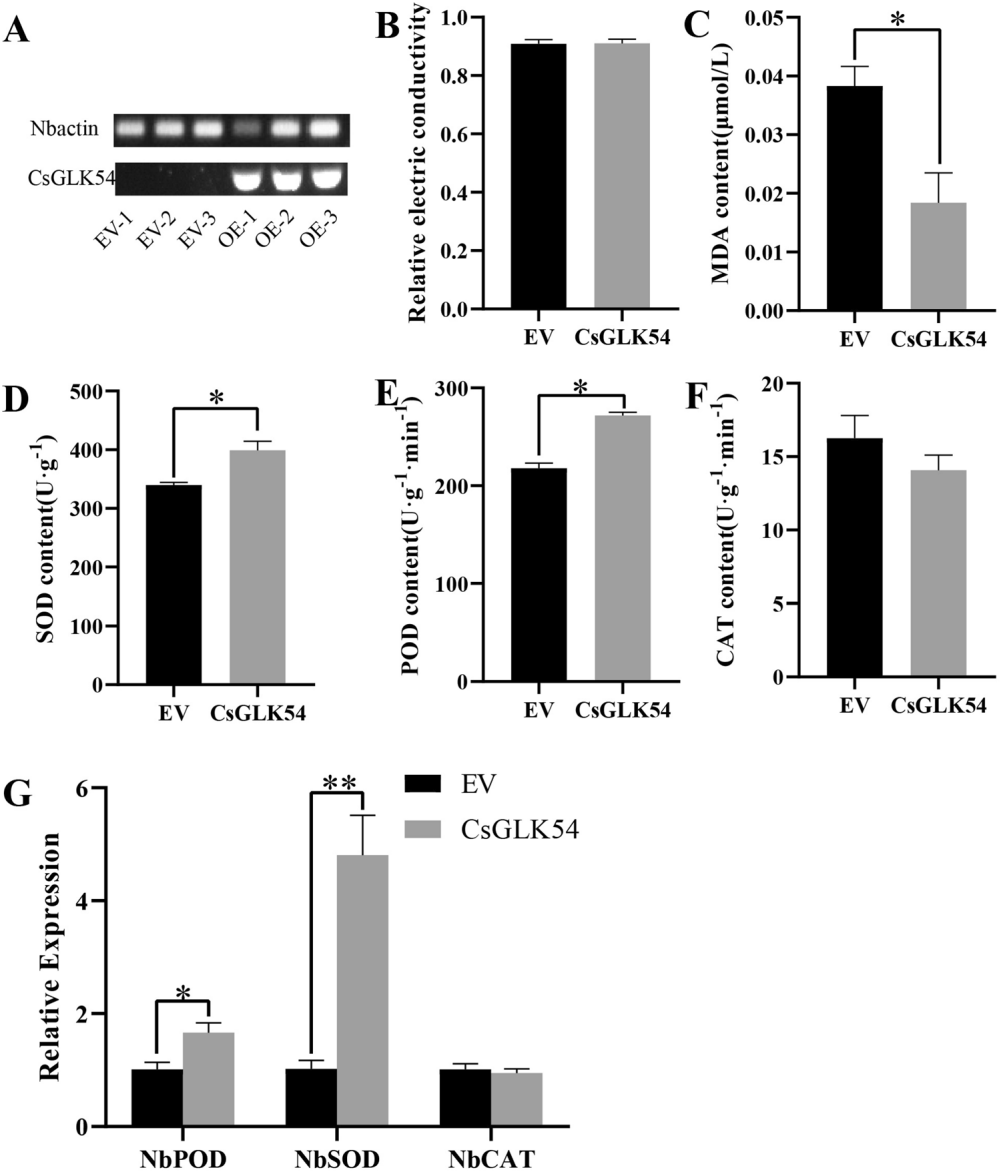
Validation of the transient conversion function of CSGLK54 in this type of tobacco: (**A**) semiquantitative validation (Supplementary Fig. 10A); (**B**) relative electrical conductivity determination; (**C**) MDA content determination; (**D**–**F**) SOD, POD, and CAT enzyme content determination; (**G**) quantitative validation of NbSOD, NbPOD, and NbCAT. The data are presented as the means ± standard errors of three biological replicates. Student’s t test and significance tests were performed (*, *P* < 0.05; **, *P* < 0.01; ***, *P* < 0.001).

Therefore, *CsGLK54* was a candidate gene for functional validation. Subcellular localization revealed that the gene was expressed in the nucleus (Fig. 9), and then *CsGLK54* was transiently transformed into tobacco (Fig. 10A), which was subjected to low-temperature stress to validate its function. Preliminary validation revealed that there was no significant change in conductivity before or after treatment, but malondialdehyde was significantly decreased (Fig. 10B and Fig. 10C). To further verify its function, we also measured the POD, SOD and CAT enzyme contents before and after treatment, and the results showed that the POD and SOD enzyme contents increased significantly (Fig. 10D and Fig. 10E), the CAT enzyme content decreased (Fig. 10F), and the expression of related genes such as *NbPOD* and *NbSOD* increased significantly in tobacco, while there was no significant change in *NbCAT* before or after treatment (Fig. 10G). Combined with the validation results (Table S6), we can preliminarily determine that *CsGLK54* regulates low-temperature stress and may also affect the expression of POD and SOD enzymes genes in plants thereby further regulating low-temperature stress.

## Discussion

The GOLDEN2-LIKE (GLK) transcription factor belongs to the GARP family of Myb transcription factors. Initially, identified in maize, it is widely believed to be associated with chloroplast development^30,31^. GLK family members have been found in various plantae, such as *Populus trichocarpa* (55)^32^, *Gossypium hirsutum* (146)^33^, *Foxtail Millet* (59)^34^, *Arabidopsis thaliana* (40), *Phyllostachys edulis* (78)^35^, and *Glycine max* (130)^36^, and have been shown to respond to low-temperature stress. In comparison, the tea plant GLK transcription factor family consists of 66 members, which is greater than the number found in *Populus trichocarpa*, *Foxtail Millet* and *Arabidopsis thaliana*. This difference may be due to the relatively large genome of tea plants or a significant number of gene duplication events during their evolutionary process. Gene duplication is a major source of genetic novelty, evolution, and adaptation in species. In this study, a total of 52 assembled genes of the tea plant GLK transcription factor were identified, which were unevenly distributed across 14 chromosomes. Clusters of genes were observed on chromosomes Chr06 and Chr10, which facilitated duplication events. The replication event involved 17 pairs of genes with fragmental duplications and one pair of genes with tandem duplications. These duplication events were primarily concentrated on Chr06 and Chr10. To further analyze the evolution and differentiation time of tea plant *CsGLKs*, the Ka/Ks ratios of the replicated genes were analyzed. Interestingly, all the Ka/Ks ratios were less than 1, indicating negative purifying selection. In general, gene replication events have led to the acquisition of new functions due to decreased evolutionary selection^37,38^. This suggests that all genes underwent negative purification through selection during the process of evolution, resulting in the development of new functions^39^. Since the exact timing of the evolutionary onset of tea plants is unknown, it is difficult to determine whether the evolution of these genes influenced the development of the assessed species. However, for the *CsGLK* genes, the oldest gene duplication event occurred approximately 127.72 million years ago, and we can observe the evolutionary traces of these genes from that point onward.

GLK transcription factors are activated by the N-terminal region of the heterologous system. The GLK gene consists of two conserved structural domains: the Myb-DNA-binding structural domain (HLH motif) and the C-terminal structural domain^40^. The HLH DNA-binding domain is also highly conserved^41^. In this study, we compared multiple sequences and found that the second helix region of the *CsGLK* gene (VK/VASHLQ) is highly conserved, while the first helix contains the initial sequence of PELHRR. The L and H residues are also highly conserved. It is not exactly the same as that of tobacco and citrus. These findings suggest that the evolutionary process of tea plant *CsGLKs* has led to the development of critically important or even irreplaceable functions in tea plants. Therefore, it can be concluded that tea plant *CsGLKs* are highly conserved throughout evolution, indicating their crucial role in tea plants. However, this finding does not imply that tea plant genes have not undergone any evolution. The amino acid sequences of the GLK genes are conserved across different species. However, the regulatory elements specific to each species play a role in regulating the expression of these genes, leading to differentiation and species specificity during plant evolution^42^. In this analysis of the GLK family genes in tea plants, motif 1 and motif 2 were identified as Myb-SHAQKYF, motif 3 as Myb_CC_LHEQLE, and motif 4 and motif 5 as REC_typeB_ARR-like. The functional annotation of other motifs is currently unavailable. This suggests that genes with different structures in these domains encode distinct proteins, indicating functional diversity during evolution^43^. Interestingly, *CsGLKs* were categorized into seven sections based on motif type and evolutionary classification. The exon‒intron structure of the tea plant GLK genes revealed a complex gene structure with a variable number of introns ranging from 0 to 11. The presence of introns can enhance gene expression^44^. Additionally, the promoter structure and number were similar and conserved across different subfamilies, indicating that structural changes occurred during gene evolution.

*Cis*-elements in gene promoters have been shown to be essential for plant physiological responses and environmental stresses^45^. Among the *CsGLKs*, *cis*-acting elements have been categorized into three main groups: hormone-responsive, stress-responsive and plant development-related; the hormone response *cis*-acting elements are the most common, followed by stress response *cis*-acting elements in tea plants; the promoter regions of genes responding to both abiotic stresses under drought and high salt stresses have more ABREs than do those of genes responding exclusively to a single stress^46^; and some of the genes of the *CsGLKs* contain greater numbers of ABRE and ARE *cis*-acting elements, such that all of these genes are potentially involved in the regulation of abiotic stresses in plants. In addition, the transcript abundance expression of *CsGLK54* among the candidate genes was significantly greater than that of the other genes during the low-temperature treatment stage, as shown in the heatmap, and the qRT‒PCR analysis also suggested that the expression increased with the prolongation of low-temperature treatment, which attracted our interest; subsequently, we also performed transient overexpression with low-temperature treatment. The results preliminarily confirmed our hypothesis that *CsGLK54* was more resistant to low-temperature stress, and the transgenic plants exhibited a significant increase in POD and SOD enzyme activity and a significant decrease in MDA content under low-temperature stress, which indicated that *CsGLK54* was induced by low temperature to positively regulate low-temperature stress.

## Conclusion

In this study, 66 members of the GLK gene family were first identified in tea plants and were categorized into seven groups by phylogenetic analysis and motif characterization. Phylogenetic analysis and motif characterization confirmed not only the typical structural features of tea plant GLK gene family members but also the functional diversity of *CsGLKs*. Combined with the replication events of some genes and the Ka/Ks ratio analysis of the replication events, these results showed that tea plant GLK genes not only expanded within the genome but also underwent negative purification selection. Further analysis of promoter *cis*-elements revealed that the promoter of the tea plant GLK gene contains a large number of *cis*-acting elements related to plant hormones and stress resistance. Moreover, we found that *CsGLK54* may play an important regulatory role under low-temperature stress based on transcription analysis and qRT‒PCR analysis and that the gene was expressed in the nucleus. We transiently transformed *CsGLK54* into tobacco to verify its function and found that *CsGLK54* functions as a positive regulator of low-temperature stress, which provided a foundation for further analysis of the mechanism of low-temperature stress regulation in tea plants.

### Supplementary Information

Supplementary Information.

## Data Availability

The datasets used and/or analyzed during the current study are available from the corresponding author upon reasonable request.
