# Supplementary material for: Identification and expression analysis of the GLK gene family in tea plant (Camellia sinensis) and a functional study of CsGLK54 under low-temperature stress
Source: Sci Rep. 2024 May 30;14:12465. doi: 10.1038/s41598-024-63323-1 (PMC11139860; doi:10.1038/s41598-024-63323-1)
Supplement: Supplementary file 1 — Supplementary Information. [file 41598_2024_63323_MOESM1_ESM.zip › Supplementary Fig. 10A.pdf]

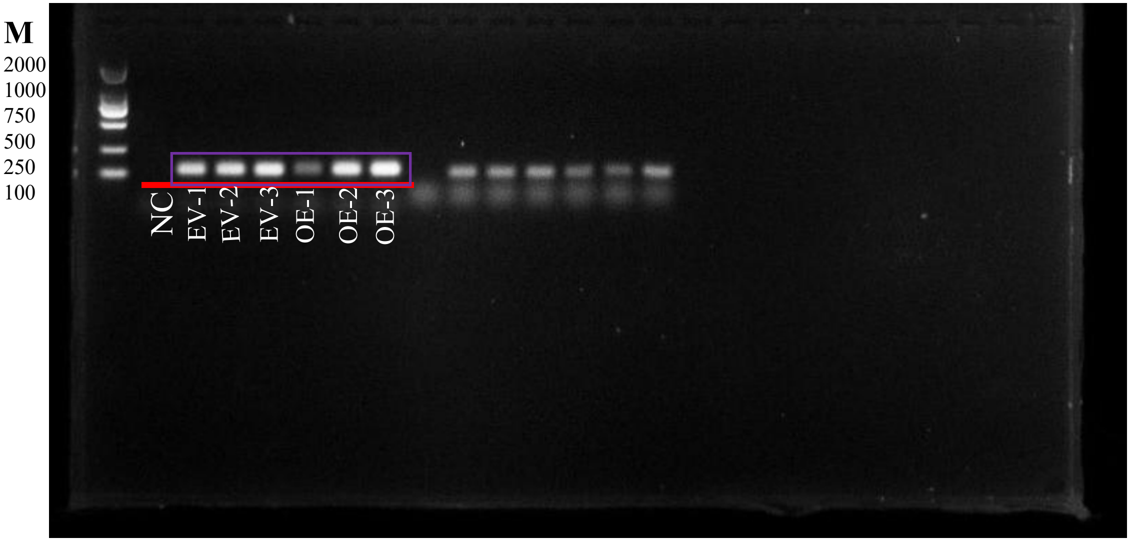

NC:negative control
EV:empty vector
OE:over-expression
The purple square area: The area is used for the test result screenshot.

Supplementary Fig. 10 (A) Original Image-1(Quality verification of transgenic tobacco templates)

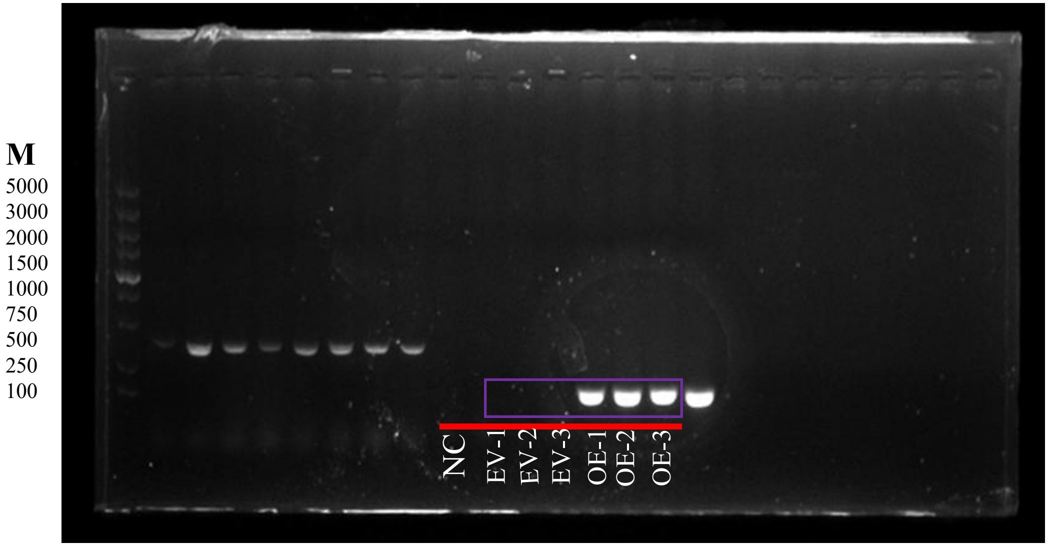

NC:negative control
EV:empty vector
OE:over-expression
The purple square area: The area is used for the test result screenshot.

Supplementary Fig. 10 (A) Original Image-2(Validation of transgenic tobacco overexpression)
