# Supplementary material for: Identification and expression analysis of the GLK gene family in tea plant (Camellia sinensis) and a functional study of CsGLK54 under low-temperature stress
Source: Sci Rep. 2024 May 30;14:12465. doi: 10.1038/s41598-024-63323-1 (PMC11139860; doi:10.1038/s41598-024-63323-1)
Supplement: Supplementary file 1 — Supplementary Information. [file 41598_2024_63323_MOESM1_ESM.zip › Supplementary tables legend.doc]

**Table S1.** FPKM values of the low-temperature transcripts of tea plant GLKs.

**Table S2.** The primers used for this study.

**Table S3.** Physical and chemical properties of GLK proteins in *C. sinensis*.

**Table S4.** Structural classification of tea plant GLK gene family members.

**Table S5.** Expression levels of eight candidate genes under low-temperature stress.

**Table S6.** Levels of 5 physiological indicators associated with low-temperature treatment and qRT‒PCR validation of genes related to enzyme activity.
